# Structure of the Monkeypox virus profilin-like protein A42R reveals potential functional differences from cellular profilins

**DOI:** 10.1107/S2053230X22009128

**Published:** 2022-09-26

**Authors:** George Minasov, Nicole L. Inniss, Ludmilla Shuvalova, Wayne F. Anderson, Karla J. F. Satchell

**Affiliations:** a Department of Microbiology–Immunology, Northwestern University Feinberg School of Medicine, Chicago, IL 60611, USA; b Center for Structural Genomics of Infectious Diseases, Northwestern University Feinberg School of Medicine, Chicago, IL 60611, USA; c Department of Pharmacology, Northwestern University Feinberg School of Medicine, Chicago, IL 60611, USA; d Department of Biochemistry and Molecular Genetics, Northwestern University Feinberg School of Medicine, Chicago, IL 60611, USA; Osaka University, Japan

**Keywords:** monkeypox, poxviruses, profilin, profilin-like protein A42R, actin, X-ray structure, emerging infectious diseases, Center for Structural Genomics of Infectious Diseases

## Abstract

The infectious disease human monkeypox is spreading rapidly in 2022, causing a global health crisis. The genomics of Monkeypox virus (MPXV) have been extensively analyzed and reported, although little is known about the virus-encoded proteome. In particular, there are no reported experimental MPXV protein structures other than computational models. Here, a 1.52 Å resolution X-ray structure of the MPXV protein A42R, the first MPXV-encoded protein with a known structure, is reported. A42R shows structural similarity to profilins, which are cellular proteins that are known to function in the regulation of actin cytoskeletal assembly. However, structural comparison of A42R with known members of the profilin family reveals critical differences that support prior biochemical findings that A42R only weakly binds actin and does not bind poly(l-proline). In addition, the analysis suggests that A42R may make distinct interactions with phosphatidylinositol lipids. Overall, the data suggest that the role of A42R in the replication of orthopoxviruses may not be readily determined by comparison to cellular profilins. Furthermore, these findings support the need for increased efforts to determine high-resolution structures of other MPXV proteins to inform physiological studies of the poxvirus infection cycle and to reveal potential new strategies to combat human monkeypox should this emerging infectious disease with pandemic potential become more common in the future.

## Abstract 2

The structure of the Monkeypox virus protein A42R has been determined at a resolution of 1.52 Å. This protein has a backbone structure similar to that of cellular profilin, but structural variation in loop regions and a surface basic patch support biochemical data showing that this protein has distinct binding interactions with actin and phosphatidylinositol lipids and is not likely to bind proline-rich domain proteins or microtubules.

## 1. Introduction

Monkeypox virus (MPXV) is a poxvirus in the *Orthopoxvirus* genus. The virus is closely related to other human pathogens, including Variola major virus (VARV), which causes smallpox, Cowpox virus (CPXV) and Vaccinia virus (VACV) (Petersen *et al.*, 2019 ▸). MPXV was first described in 1958 during an outbreak among macaques originating from Singapore that were imported into Denmark for polio-vaccine research (Parker & Buller, 2013 ▸). The disease human monkeypox is typified by headache, fever and flu-like symptoms, with characteristic pox lesions appearing shortly after symptom onset (McCollum & Damon, 2014 ▸; Durski *et al.*, 2018 ▸). Although most patients resolve the infection in two to four weeks, the disease can be fatal, with case-fatality ratios of 10.6% for infections by MPXV clade 1 strains (formerly known as the Central African clade) and 3.6% for infections by MPXV clade 2 and 3 strains (formerly known as the West African clade) (Bunge *et al.*, 2022 ▸).

Human monkeypox is most commonly a zoonotic infection contracted from wild animals and passed from human to human by direct contact (Bunge *et al.*, 2022 ▸). In the rainforest areas of Western and Central Africa discrete outbreaks have historically occurred, impacting only a small number of patients, except in the Democratic Republic of Congo, which has reported more than 1000 cases per year since 2005 (Durski *et al.*, 2018 ▸; Bunge *et al.*, 2022 ▸). Previously, the largest outbreak outside Western and Central Africa occurred in 2003, when 47 cases in the Midwestern United States were linked to contact with prairie dogs from a distributor that also imported rodents from Ghana (Centers for Disease Control and Prevention, 2003 ▸; Reynolds *et al.*, 2006 ▸). The now largest ongoing outbreak of MPXV began in April 2022 and is linked to more than 57 000 cases across 103 countries, predominantly in the United States, Europe and Brazil (Centers for Disease Control and Prevention, 2022 ▸). The World Health Organization has declared human monkeypox a public health emergency of global concern (World Health Organization, 2022 ▸).

MPXV is an enveloped, brick-shaped virus with a linear, double-stranded DNA genome. The representative MPXV genome from isolate Zaire-96-I-16 is 196.8 kb with 191 open reading frames and derives from clade 1 (Shchelkunov *et al.*, 2001 ▸). The 2022 outbreak is a new branch on the phylogenetic tree within clade 3 and is most similar to the strain that caused a 2017–2018 outbreak in Nigeria that spread to the United Kingdom (Isidro *et al.*, 2022 ▸). More than 400 genomes of the 2022 outbreak strain are already available from the National Center for Bioinformatic Information (NCBI) GenBank, with genomes of 184.7–198.0 kb annotated to have between 143 and 214 open reading frames. Analysis of the genomes reveals that the strain is similar to isolates from West Africa and differs from the 2018–2019 isolates by 50 single-nucleotide polymorphisms (Isidro *et al.*, 2022 ▸).

**Figure 1:**
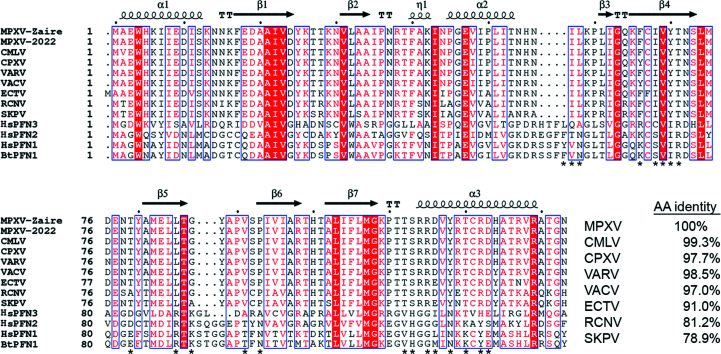
Sequence alignment of MPXV A42R with orthopoxvirus profilin-like proteins and mammalian profilin proteins. Abbreviations are as described in Section 2. Asterisks below the *Bt*PFN1 sequence indicate residues that form primary contacts with actin (Schutt *et al.*, 1993 ▸).

Despite extensive knowledge of the genomics and epi­demiology of MPXV, the proteome of MPXV has not been well studied. There are no structures of proteins encoded by the MPXV genome other than computational models. The A42R protein that is encoded by the gp153 locus of MPXV has significant amino-acid sequence identity to eukaryotic cell profilin proteins (Van Vliet *et al.*, 2009 ▸; Fig. 1 ▸). Proteins from the profilin family are actin-binding proteins that participate in the assembly and regulation of F-actin (Witke, 2004 ▸; Krishnan & Moens, 2009 ▸; Davey & Moens, 2020 ▸). The VACV profilin-homology protein (VACV A42R), which is 98% identical to MPXV A42R (Fig. 1 ▸), is a late-expressing viral protein with a weak affinity for actin (Blasco *et al.*, 1991 ▸; Machesky *et al.*, 1994 ▸). A peptide from VACV A42R (^88^YAPVSPIVI^96^) is a CD8+ T-cell epitope (Moutaftsi *et al.*, 2006 ▸). Genetic knockout of the open reading frames for the profilin-homology proteins of VACV and CPXV demonstrated these proteins are not essential for the replication of poxviruses *in vitro* (Blasco *et al.*, 1991 ▸; Xu *et al.*, 2014 ▸). Thus, the role of these highly conserved profilin-like proteins in the poxvirus infection cycle is unknown. Here, we report the structure of the profilin-like protein A42R, the first structure of an MPXV protein to be deposited in the Protein Data Bank (PDB). Although the structure detailed here was first deposited and released in 2014, it remains the only protein from MPXV in the PDB and the only poxvirus profilin-like protein with a known structure. Comparison with structures of human and bovine profilin proteins reveals significant differences in functional regions, indicating that the role of MPXV A42R in the viral life cycle may not be easily determined based solely on our understanding of cellular profilin function.

## 2. Materials and methods

### 2.1. Protein expression, purification and crystallization

The open reading frame encoding A42R was amplified from genomic DNA of the MPXV Zaire-96-I-16 strain (NCBI accession NC_003310.1) into vector pMCSG53 (Stols *et al.*, 2002 ▸). Recombinant A42R was expressed in and purified from *Escherichia coli* BL21(DE3)-Magic cells according to published methods (Shuvalova, 2014 ▸). The purified protein was set up for crystallization at 2.43 mg ml^−1^ in 0.5 *M* sodium chloride, 0.01 *M* Tris–HCl buffer pH 8.3, 5 m*M* β-mercapto­ethanol as 2 µl crystallization drops (1 µl protein solution and 1 µl reservoir solution) in 96-well crystallization plates using commercially available screens. Diffraction-quality crystals were obtained from 0.1 *M* MMT buffer pH 9.0, 25%(*w*/*v*) PEG 1500 (condition 42 of the PACT screen from Qiagen).

### 2.2. Structure determination and refinement

Diffraction data were collected on the 21-ID-G beamline of the Life Science Collaborative Access Team (LS-CAT) at the Advanced Photon Source, Argonne National Laboratory, USA. The data set was processed and scaled using the *HKL*-3000 suite (Minor *et al.*, 2006 ▸). The structure was solved by the single-wavelength anomalous dispersion (SAD) method using selenomethionine-derivatized protein. The initial solution went through several rounds of refinement in *REFMAC* version 5.7.0032 (Murshudov *et al.*, 2011 ▸) and manual model correction using *Coot* (Emsley *et al.*, 2010 ▸). The water molecules were generated using *ARP*/*wARP* (Cohen *et al.*, 2008 ▸) and ligands were added to the model manually during visual inspection in *Coot*. Translation–libration–screw (TLS) groups were created by the *TLSMD* server (http://skuld.bmsc.washington.edu/~tlsmd/; Painter & Merritt, 2006 ▸) and TLS corrections were applied during the final stages of refinement. *MolProbity* (http://molprobity.biochem.duke.edu/; Chen *et al.*, 2010 ▸) was used to monitor the quality of the model during refinement and for final validation of the structure. Structure analysis and figure preparation was performed with *UCSF ChimeraX* (Pettersen *et al.*, 2021 ▸).

### 2.3. Sequence analysis

Sequence analysis was performed using sequences from MPXV Zaire-96-I-16 (MPXV-Zaire, NCBI accession NP_536579.1), MPXV-2022 clade 3 (MPXV-2022, YP_010377149.1), Camelpox virus (CMLV, Q775N7.1), Cowpox virus (CPXV, NP_619961.1), Variola major virus (VARV, ABF24320.1), Vaccinia virus (VACV, QQ05880.1), Ectromelia virus (ECTV, NP_671660.1), Raccoonpox virus (RCNV, YP_009143471.1), Skunkpox virus (SKPV, YP_00928256.1), *Homo sapiens* (human) profilin-1 (*Hs*PFN1, NP_005013.2), human profilin-2 (*Hs*PFN2, NP_444252.1), human profilin-3 (*Hs*PFN3, NP_001025057.1) and *Bos taurus* (bovine) profilin-1 (*Bt*PFN1, NP_001015592.1). The alignment was prepared using *Clustal Omega* (Sievers & Higgins, 2021 ▸) and *ESPript*3 (Robert & Gouet, 2014 ▸).

### 2.4. Data availability

The final models and diffraction data were deposited in the Protein Data Bank (https://www.wwpdb.org/) as PDB entry 4qwo.

## 3. Results

### 3.1. MPXV A42R resembles human profilins

The open reading frame encoding MPXV A42R was amplified and cloned from MPXV Zaire-96-I-16 strain DNA (NCBI accession NP_536579.1; Shchelkunov *et al.*, 2001 ▸). The A42R protein was expressed in *E. coli* in selenomethionine-containing medium and purified as described in Section 2. Diffraction-quality crystals grew from 25%(*w*/*v*) PEG 1500, 0.1 *M* MMT buffer pH 9.0 and belonged to space group *P*2_1_2_1_2_1_. The structure was determined using the SAD method and was refined to 1.52 Å resolution (Table 1 ▸).

**Figure 2:**
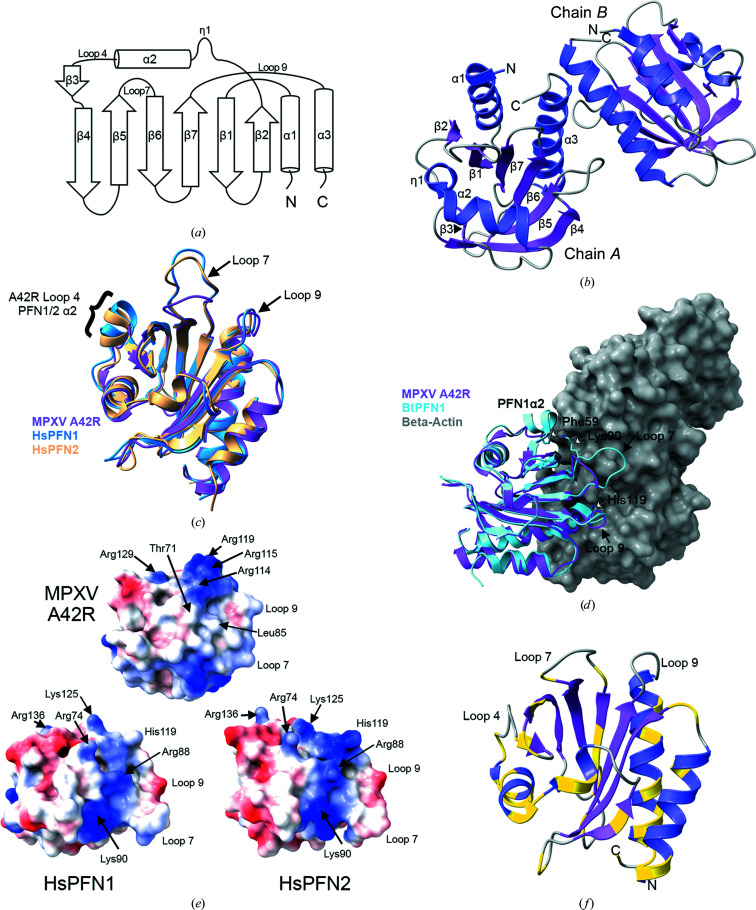
Structural analysis of MPXV A42R. (*a*) Schematic of the β-strand and α-helical arrangement of the A42R protein. (*b*) A cartoon representation of the X-ray structure of the MPXV-Zaire A42R structure (PDB entry 4qwo) with β-strands in violet and α-helices in dark purple. Strands and helices in chain *A* are marked as indicated in (*a*). (*c*) Overlay of MPXV A42R (violet) with *Homo sapiens* (human) *Hs*PFN1 (blue; PDB entry 1fil; A. A. Fedorov, T. D. Pollard & S. C. Almo, unpublished work) and human *Hs*PFN2 (beige; PDB entry 1d1j; Nodelman *et al.*, 1999 ▸). Loops are labeled according to the MPXV A42R structure as shown in (*a*). (*d*) Overlay of MPXV A42R (violet) with the structure of *Bos taurus* (bovine) *Bt*PFN1 (light blue) bound to β-actin (gray; PDB entry 3ub5; Porta & Borgstahl, 2012 ▸). Loops are labeled according to the MPXV A42R structure as shown in (*a*). (*e*) Electrostatic surface projections of MPXV A42R, *Hs*PFN1 and *Hs*PFN2 (PDB entries 4qwo, 1fil and 1d1j, respectively). Negatively charged residues are shown in red and positively charged residues are in blue, with residues noted in the text indicated. Loops as indicated in (*d*) are shown for orientation of the actin-binding region. (*f*) Structure of MPXV A42R oriented as in (*c*) and colored as in (*b*), with residues that vary in any of the nine orthopoxviruses in the alignment in Fig. 1 ▸ indicated in yellow. Loops are as marked as in (*a*).

The asymmetric unit consisted of two polypeptide chains. Full-length chain *A* was intact across all 133 residues of A42R and also contained an N-terminal alanine residue originating from the Tobacco etch mosaic virus (TEV) protease recog­nition site. Chain *B* contained only residues 2–133. The overall structure formed a seven-stranded antiparallel β-sheet surrounded by three α-helices and one partial helix (Figs. 2 ▸ *a* and 2 ▸ *b*). A *DALI* search for protein structures with a similar fold (Holm, 2022 ▸) revealed that MPXV A42R closely resembles structures determined for the ubiquitously expressed human profilin-1 (PFN1; 32% identity) and the neuronal specific profilin-2 (PFN2; 26% identity), which share a well conserved fold (Krishnan & Moens, 2009 ▸). There is also weaker structural homology to *Arabidopsis thaliana* profilin-3 (PFN3; 23% identity), which also functions in actin dynamics (Qiao *et al.*, 2019 ▸). Therefore, our structure represents a family of profilin-like orthopoxviral proteins that may play a role in altering actin dynamics during viral replication in cells.

When A42R was overlaid with human PFN1 (PDB entry 1fil; A. A. Fedorov, T. D. Pollard & S. C. Almo, unpublished work) and PFN2 (PDB entry 1d1j; Nodelman *et al.*, 1999 ▸), the backbones of the structures aligned with a root-mean-square deviation (r.m.s.d.) of 0.8 Å (117 pruned C^α^ pairs) for PFN1 and an r.m.s.d. of 1.0 Å (110 pruned C^α^ pairs) for PFN2. The major differences between the structures are located in the loop regions (Fig. 2 ▸ *c*). In particular, loop 4 between α2 and β3 in A42R from MPXV is four residues shorter than the same loop in PFN1 and PFN2. In the human profilin proteins, part of this loop forms a helix (PFN1/PFN2 α3). Thus, MPXV A42R has only three helices surrounding the seven-stranded β-sheet, compared with the four helices that typify other profilin proteins. In addition, loop 7 between strands β5 and β6 has a three-residue deletion that makes this loop shorter. Interestingly, this loop is also shorter in human PFN3 than in PFN1 and PFN2 (Fig. 1 ▸), although the impact of this on its function is not known. Structural analysis of the computational model of the A42R homolog from Ectromelia virus (ECTV) also predicted significant differences in these loops compared with human profilins (Butler-Cole *et al.*, 2007 ▸).

### 3.2. Analysis of the MPXV A42R structure supports the weak binding of poxvirus profilin-like proteins to actin

Profilins interact directly with actin to sequester actin monomers and then deliver the ATP–actin complex to the growing end of an actin filament (Davey & Moens, 2020 ▸). Biochemical studies have shown that VACV A42R binds actin, but the binding affinity is weak compared with both PFN1 and PFN2 (Machesky *et al.*, 1994 ▸). Comparative analysis of the X-ray structure of MPXV A42R with those of mammalian PFN1 and PFN2 suggests that the shortening of these loop regions could have significant functional impact on the binding affinity of MPXV A42R for actin. An overlay of the structure of A42R from MPXV with the structure of *Bos taurus* (bovine) PFN1 (*Bt*PFN1) with bound β-actin (PDB entry 3ub5; Porta & Borgstahl, 2012 ▸) shows that loops 4, 7 and 9 participate in the formation of the patch on the surface that interacts with the surface of actin (Fig. 2 ▸ *d*). However, the residues involved in this critical interaction are different in A42R. In particular, amino-acid differences in loop 9 of A42R eliminated the critical interaction of PFN1 His119 with a hydrophilic pocket of the β-actin formed in part by Tyr133 and Tyr169 (Fig. 2 ▸ *d*). Loss of this histidine interaction with actin may in part be replaced by weak hydrogen-bond interactions between Arg115 and Arg119 of MXPV A42R and Glu61 of actin. Further, Phe59 in α3 of PFN1 forms a stacking interaction with His173 of actin. This critical Phe residue is lost in the replacement of α3 in *Bt*PFN1 with the shorter loop 4 in MPXV A42R. Finally, Lys90 in loop 7 of PFN1 interacts with Asp288 of actin, but this residue is missing in MPXV A42R due to the deletion in loop 7. In total, 19 out of 20 *Bt*PFN1 residues that form the primary contact interface with actin are substituted in MPXV A42R (Fig. 1 ▸).

### 3.3. The PIP2 interaction surface is distinct in MPXV A42R compared with profilins

Profilins are known to regulate actin polymerization through interaction with phosphatidylinositol-(4,5)-bisphos­phate (PIP2) at cell membranes. PIP2 binding to profilins also sequesters PIP2 from turnover by phospholipase C-γ (Davey & Moens, 2020 ▸). PIP2 binding to human profilins is proposed to require a positively charged surface that involves residues Arg74, Arg88, Lys90 and Lys125 (Fig. 2 ▸ *e*) and that partially overlaps the actin-binding face (Fedorov *et al.*, 1994 ▸; Krishnan & Moens, 2009 ▸). Comparison of electrostatic projections of the proposed PIP2 binding surface shows that MPXV A42R has a significant reorganization of basic and hydrophobic regions compared with PFN1 and PFN2 (Fig. 2 ▸ *e*). In particular, mutagenesis studies demonstrated that Arg88 in PFN1 and surrounding residues are critical for PIP2 binding (Sohn *et al.*, 1995 ▸; Krishnan & Moens, 2009 ▸). In MPXV A42R, Arg88 of PFN1 is replaced by Leu85 and the nearby Lys90 of PFN1 is lost because of the deletion in loop 7. Further, Arg74 in PFN1 is changed to a threonine (Thr71) in MPXV A42R. Thus, the surface in this region is hydrophobic rather than basic. Biochemical studies have nonetheless shown that MPXV A42R does bind PIP2 (Gieselmann *et al.*, 1995 ▸). Structural analysis of MPXV A42R suggests that PIP2 may bind via an arginine patch comprised of Arg114, Arg115 and Arg119. This suggestion is consistent with a role of the C-terminal α-helix of PFN1, and specifically of Lys125 (which is in the same position as Arg119 in MPXV A42R), as a contributor to PIP2 binding (Sohn *et al.*, 1995 ▸; Krishnan & Moens, 2009 ▸). It has also been suggested that Arg136 contributes to a second PIP2 binding site in PFN1 (Lambrechts *et al.*, 2002 ▸). This residue corresponds to Arg129 in MPXV A42R and interestingly is conserved in all profilin and viral profilin-like proteins (Fig. 1 ▸), indicating that this PIP2 binding site may be conserved in the viral profilin-like proteins.

### 3.4. Possible interactions of MPXV A42R with other proteins

In addition to their interaction with actin and PIP2, profilins bind to actin-associated proteins and other cellular proteins (Kaiser & Pollard, 1996 ▸; Witke, 2004 ▸; Davey & Moens, 2020 ▸). Many of these interactions are through the poly(l-proline) region formed by the N- and C-terminal helices, and the binding of proteins with proline-repeat domains is regulated by the phosphorylation of a serine located on the C-terminal helix (Davey & Moens, 2020 ▸). However, VACV A42R does not bind poly(l-proline) (Machesky *et al.*, 1994 ▸), although the poxvirus profilin-like proteins do have a threonine (Thr131) at the position of the phosphorylated serine. Thus, it is unlikely that MPXV A42R binds to proteins with proline-repeat domains despite the presence of Trp4 (Trp5 in VACV A42R), which is known to disrupt binding to poly(l-proline)-containing proteins when changed by site-directed mutagenesis (Kaiser & Pollard, 1996 ▸).

PFN1 is also known to interact with microtubules through Met114 and Gly118 (Henty-Ridilla *et al.*, 2017 ▸). As noted above, in MPXV A42R these residues are changed to form an arginine patch and we posit that this patch may contribute to PIP2 binding. Since the surface at this face is dramatically changed in MPXV A42R, we speculate that this protein may not bind microtubules.

Finally, ECTV A42R has been shown to bind and co-localize with cellular tropomyosin, a protein that polymerizes along actin filaments to regulate the binding of actin-associated proteins to the filaments (Butler-Cole *et al.*, 2007 ▸). Tropomyosin has not been recognized as a ligand of human profilins (Witke, 2004 ▸; Davey & Moens, 2020 ▸). Although the regions of the ECTV A42R protein that bind tropomyosin are not yet known, this finding indicates that poxvirus profilin-like proteins may engage in unique protein–protein interactions and regulate actin in a distinct manner from human profilins.

### 3.5. The structure of A42R is highly conserved across the orthopoxviruses

To understand whether the structure reported here would impact our understanding of other poxviruses, we compared the sequence of MPXV A42R with those of other poxvirus profilin-like proteins. MPXV A42R differs from VARV and VACV A42R by only two amino acids and from CPXV A42R by three amino acids (Fig. 1 ▸). Sequence alignment with other more distant orthopoxviruses also shows high identity across all residues, with Skunkpox virus (SKPV) A42R being the most distant homolog, with 79% identity. In total, we identified 39 single-residue differences across nine homologous orthopoxvirus A42R proteins and mapped them onto our structure (Fig. 2 ▸ *f*). These differences span the entire protein and are, in general, modest substitutions with retention of residue characteristics. Interestingly, the residues that form β1 and the neighboring antiparallel strand β7 are 100% identical across all nine homologs, suggesting a key role for this region in protein stability and/or function. All orthopoxvirus profilin-like proteins also have loops 4 and 7 that are shorter compared with cellular profilins. Further, the presence of all three arginine residues that form the arginine patch in MPXV is also conserved across the orthopoxviral proteins, except for SKPV, in which Arg119 is substituted by an asparagine residue. The conservation of the potential functional elements suggests that the structure of MPXV A42R is a valid homologous representative across the orthopoxvirus genus.

## 4. Discussion

Overall, our structure of MPXV A42R is not inconsistent with the hypothesis that this protein could function to regulate actin remodeling, similar to the host profilins. However, closer structural comparisons reveal that A42R has significant differences that may impact its interactions with other proteins or ligands and that could indicate distinct functional differences of this protein in cells. As most biochemical studies of A42R were conducted in the 1990s, our work highlights the value of further investigation of the function of A42R and other viral profilin-like proteins in the viral life cycle. New molecular and biochemical studies are needed to determine the biologically relevant binding partners of A42R and the role of these interactions during viral replication in cells.

Although the VACV and CPXV profilin-like proteins were not essential for viral replication in cultured cells (Blasco *et al.*, 1991 ▸; Xu *et al.*, 2014 ▸), it remains possible that this protein is important during infection in different cell types or in a clinical setting. Neither the VACV nor CPXV strains with the open reading frames for the profilin-like proteins interrupted were tested in animals, and thus the impact of the protein on viral replication *in vivo* remains unknown. Further, our analysis of the first structurally characterized protein from MPXV suggests that there would be value in conducting structural studies of other MPXV proteins, even those that may be similar to host proteins with known structures. Indeed, this may be critical to reveal more about the physiology of MPXV if the current outbreak continues to spread or if outbreaks of similar scope begin to recur more frequently. Such efforts could inform the development of new thera­peutics. Currently, only tecovirimat, an envelope protein wrapping inhibitor, and brincidofovir, a nucleoside analog, are FDA-approved for the treatment of monkeypox (Delaune & Iseni, 2020 ▸). Structural modeling may serve to fill a knowledge gap in the absence of X-ray structures. A recent preprint reported an analysis of the genome of the newly circulating strain of MPXV and predicted the 3D structures of the proteins encoded by 123 different open reading frames (Parigger *et al.*, 2022 ▸). However, for structure-guided drug discovery and the establishment of structure–activity relationships, high-resolution crystal structures such as the 1.52 Å resolution structure reported here are still of high value to conduct advanced biological and translational studies of this understudied pathogen.

## Supplementary Material

PDB reference: A42R profilin-like protein from Monkeypox virus, 4qwo

## Figures and Tables

**Table 1:** Data-quality and refinement statistics Values in parentheses are for the outer shell.

PDB code	4qwo
Data collection	
Wavelength (Å)	0.97856
Temperature (K)	100.0
Detector	MarMosaic 300 mm CCD
Crystal-to-detector distance (mm)	170.0
Oscillation per image (°)	0.6
Oscillation range (°)	180.0
Mosaicity (°)	0.23–0.31
Space group	*P*2_1_2_1_2_1_
*a*, *b*, *c* (Å)	41.62, 50.50, 119.62
α, β, γ (°)	90.00, 90.00, 90.00
Resolution range (Å)	30.00–1.52 (1.55–1.52)
Total No. of reflections	282411 (14076)
No. of unique reflections	39686 (1955)
*R*_merge_ (%)	8.7 (54.7)
*R*_p.i.m._ (%)	3.5 (21.8)
CC_1/2_	0.996 (0.904)
Completeness (%)	100.0 (100.0)
〈*I*/σ(*I*)〉	31.4 (3.2)
Multiplicity	7.1 (7.2)
Wilson *B* factor (Å^2^)	14.3
Refinement	
Resolution range (Å)	28.81–1.52 (1.56–1.52)
Completeness (%)	99.9 (99.0)
No. of reflections	37630 (2843)
*R*_work_/*R*_free_ (%)	14.1/16.6 (17.0/20.2)
Protein chains/atoms	2/2302
Ligand/solvent atoms	42/478
Mean temperature factor (Å^2^)	16.3
Coordinate deviations	
R.m.s.d., bond lengths (Å)	0.010
R.m.s.d., angles (°)	1.517
Ramachandran plot	
Favored (%)	97.0
Allowed (%)	3.0
Outside allowed (%)	0.0
